# Prospective Associations between Depression and Obesity for Adolescent Males and Females- A Systematic Review and Meta-Analysis of Longitudinal Studies

**DOI:** 10.1371/journal.pone.0157240

**Published:** 2016-06-10

**Authors:** Munim Mannan, Abdullah Mamun, Suhail Doi, Alexandra Clavarino

**Affiliations:** 1 School of Pharmacy, The University of Queensland, 20 Cornwall Street, Woolloongabba, 4102, Queensland, Australia; 2 School of Population Health, The University of Queensland, Herston Road, Herston, 4006, Queensland, Australia; 3 Research School of Population Health, ANU College of Medicine, Biology and Environment, Australian National University, Acton, ACT 2601, Australia; Radboud University Medical Centre, NETHERLANDS

## Abstract

Adolescent obesity and depression are increasingly prevalent and are currently recognised as major public health concerns worldwide. The aim of this study is to evaluate the bi-directional associations between obesity and depression in adolescents using longitudinal studies. A systematic literature search was conducted using Pubmed (including Medline), PsycINFO, Embase, CINAHL, BIOSIS Preview and the Cochrane Library databases. According to the inclusion criteria, 13 studies were found where seven studies evaluated depression leading to obesity and six other studies examined obesity leading to depression. Using a bias-adjusted quality effects model for the meta-analysis, we found that adolescents who were depressed had a 70% (RR 1.70, 95% CI: 1.40, 2.07) increased risk of being obese, conversely obese adolescents had an increased risk of 40% (RR 1.40, 95% CI: 1.16, 1.70) of being depressed. The risk difference (RD) of early adolescent depression leading to obesity is 3% higher risk than it is for obesity leading to depression. In sensitivity analysis, the association between depression leading to obesity was greater than that of obesity leading to depression for females in early adulthood compared with females in late adolescence. Overall, the findings of this study suggest a bi-directional association between depression and obesity that was stronger for female adolescents. However, this finding also underscores the importance of early detection and treatment strategies to inhibit the development of reciprocal disorders.

## Introduction

Adolescence is a critical period in human life because of the multiple changes which occur during this life stage [1]. Obesity and depression are more common during this period and there is an increasing likelihood of the simultaneous occurrence of these disorders [2, 3]. This increased vulnerability to both depression and obesity in adolescence suggests a possible bi-directional association [4, 5]. There are several possible mechanisms linking depression and obesity including behavioural and lifestyle factors as well as biological and genetic factors. Adolescents, who are depressed, may change their appetite and dietary patterns resulting in weight gain or loss [6], are more inclined to favour carbohydrate rich food which provide pleasure or comfort [7], increase sedentary activity [8], are vulnerable to disordered sleeping [9], and are at greater risk of binge eating [10, 11]. These factors may lead to an increased risk of obesity. Adolescents who are obese may experience stigmatization [12, 13], poor body image and low self-esteem [14] increasing their vulnerability to depression [15]. Their behaviours and lifestyle, particularly poor dietary habits and sedentary activity in terms of reducing physical activity as well as disordered sleep may also contribute to depression [16–18]. There are also a number of shared biological mechanisms including inflammation [19, 20], impaired glycaemic control [21], dysregulation of the hypothalamic–pituitary–adrenocortical axis [22], and neuroendocrine mechanisms via leptin melanocortinergic-BDNF signalling [23] that have been implicated in the aetiology of both depression and obesity. Genetic susceptibility may also potentially link both obesity and depression [24], although this is controversial [25].

There have been two systematic reviews [26, 27] and one meta-analysis [28] using prospective or retrospective longitudinal studies that have assessed weight status and psychiatric symptoms in children and adolescents with a particular emphasis on depression. A review by Incledon et al. assessed the influence of psychological predictors during childhood and adolescence on the subsequent development of obesity. They found that depressive symptoms in obese male and female adolescents was associated with a greater than average weight gain [26]. Liem et al. also reported a positive association between depressive symptoms between ages 6 to 19 years and overweight in later life [27]. However, a meta-analysis of prospective studies found that the association between obesity and depression was greater in adolescents, particularly females [28]. The bi-directional link between obesity and depression has been assessed by two recent reviews [29, 30]. Korczak et al. found a reciprocal relationship between depression and obesity again, primarily for adolescent females. They found that obese adolescent females have a greater risk of experiencing depressive symptoms in adulthood, and that in female adolescents, depressive symptoms are associated with a higher risk of obesity in adulthood [29]. The findings reported by Muhlig et al. were mixed—three out of eight longitudinal studies reported associations between obesity and subsequent depression in female children and adolescents only, and three out of nine studies provided evidence for depression leading to obesity with two studies reporting significant results only for females and one only for male children and adolescents [30].

Altogether, the evidence from existing studies is mixed. The current reviews have compiled this evidence using data from both children and adolescents. Therefore, based on the available evidence it is difficult todetermine whether or not there is an association between depression and obesity in adolescents. In most of the studies reported the association between these two disorders is significantly modified by gender whereby females are at higher risk compared with males [31]. However, the magnitude of risk of obesity and depression for adolescent males and females is yet undetermined.

To the best of our knowledge, there are currently no systematic reviews or meta-analyses that have used data only from longitudinal studies in order to quantify the bi-directional link between obesity and depression. Moreover, previous meta-analyses have used conventional methods that do not consider the strengths and weaknesses of individual studies, therefore, these analyses have been unable to investigate the effect of various methodological deficiencies on the pooled estimates [32–34]. In addition, none of the previous studies have measured the impact (i.e. excess risk) of any associations on public health.

In this systematic review and meta-analysis, we quantitatively examine the evidence from longitudinal studies only, and attempt to provide a more rigorous assessment of the direction and magnitude of the association between obesity and depression in adolescence by gender. Here we are using more robust methods—the quality effect model (QE) developed by Doi and others [35–37]—to handle the methodological heterogeneity of the studies included in our review.

## Materials and Methods

This study was performed following the Preferred Reporting Items for Systematic Reviews and Meta-Analysis (PRISMA) statement for reporting systematic reviews and meta-analyses [38] (detailed in Table A in S1 File, PRISMA checklist).

### Search Strategy

We carried out a comprehensive, systematic, computerized literature search using Pubmed (including Medline), PsycINFO, Embase, CINAHL, BIOSIS Preview and the Cochrane Library databases for the period 1961 to January, 2015. We searched for articles examining the association between depression and obesity in adolescence. References from the relevant literature were then hand searched and used to identify additional relevant studies. The search strategy is determined by combining the following key words and medical subject headings for adolescence: depression, depressive disorder, depressive symptoms, major depression, metabolic syndrome, overweight, obesity, adiposity, body mass index (BMI), intra-abdominal fat, waist-hip ratio, and waist circumference.

### Inclusion and exclusion criteria

Using the PICOS criteria (Table B in S1 File) studies were included if they (1) were published in the English language; (2) examined the prospective association between obesity and depression or vice versa; (3) contained extractable effect estimates (for depression or obesity) overall or separately by gender; (4) had a follow-up period of at least 1 year; (5) used a community or population based sample and; (6) used standardized cut-offs for BMI [39] and assessed depression either clinically or using rating scales based on symptoms. We excluded studies that (1) were published in languages other than English; (2) were case reports, qualitative reports, comments, letters and reviews; (3) did not report information pertinent to the key clinical questions and (4) defined weight gain or loss/change using criteria other than BMI categories or in which the assessment of depression was unspecified.

### Data extraction and quality appraisal

A standard data extraction form was developed based on the STROBE statement that would summarize the study design and other relevant raw data for each article, this was completed by an independent reviewer [40]. To keep the reported data homogenous required the transformation of weights from pounds to kilograms in a few studies. Studies reporting their outcomes only in odds ratios (OR) or in relative risks (RR) were synthesised. A quality scoring instrument (Table C in S1 File) was devised based on standard bias criteria for observational studies [41] to adjust for study deficiencies. According to the standard procedure all the data abstraction, inclusion, exclusion and quality scoring was performed by two reviewers (MM and SD). Any disagreement in the scoring was resolved by consensus and each item that was not deficient was given a point. These were then summed to form a univariate quality score (Table D in S1 File).

### Statistical Analysis

Four-fold cells (**2×2** tables) were imputed for all binary point estimates using the reconstruction method [42] to convert all ORs to RRs because the OR may not adequately portray risk [43]. In addition to the RR, the contribution of risk from obesity and depression was defined using an absolute term known as the risk difference (RD). This can be used to judge the impact of the risk of obesity and depression on populations and individuals. To compute the impact of obesity and depression, the RD was defined based on an estimated cumulative incidence of the outcome at 1 year across the studies with follow-ups of more than a year. The 1-year follow-up study was considered as the base year and follow-up studies of more than 1 year were interpolated to calculate the cumulative incidence. For these studies of duration more than 1 year, the yearly incidence rate (IR) was estimated as

$$IR=-[\text{ln}(1-CI_{t})/t]$$

where *CI*_t_ is the cumulative incidence proportion of events at the end of the study and *t* is the duration of follow-up [44]. The 1 year cumulative incidence was then computed as

$$1-e^{-IR\left(1\right)}$$

where *e* is exponential of IR. Two separate analyses were conducted: one for depression and another one for obesity as outcomes respectively. Given the significant methodological heterogeneity and inconsistency among the studies included, we used the quality effects (QE) model [35–37] to perform bias adjusted analyses. The QE model is the only model currently available that allows empirical bias adjustment of observational studies. We used this in lieu of the random effects (RE) model[45] because the methodology associated with the RE model, according to authors opinion, has limitations to the extent that, even in standard meta-analyses, there is a lack of interpretation of an RE summary [46]. Moreover, use of the RE model requires assumptions that are unlikely to be valid in practice. Most notably, the RE analysis is based on the premise that the trials performed are representative of some hypothetical population of trials, and that the heterogeneity can be represented by a single variance. We take the approach in this paper that when heterogeneity has been detected, there is a strong case for investigating its possible origin, and redistributing the weights based on such a determination [47]. Heterogeneity was determined to be present when the value of *τ*^2^ was greater than zero and/or the Q-statistic was significant at a *p* < 0.10 [48]. All analyses were performed using MetaXL ver 2.0 [49].

### Sensitivity analysis and publication bias

To assess the robustness of the meta-analysis, sensitivity analyses were performed by examining the effect of altering the selection criteria of the studies included in the pooled results. Several selection criteria were examined including region of origin (US vs Europe and other countries), risk of obesity and depression in late adolescence (16 to 18 years) vs. young adulthood (over 18.5 to 35 years), follow-up duration (<10 years vs ≥10 years), number of covariates adjusted for (<5 and ≥ 5) and assessment of depression (i.e. clinically diagnosed depression vs symptom score-based assessment). Clinically diagnosed studies were identified based on the depressive disorder of the participants as defined by the authors of each study. These included depressive mood, specific syndromes or use of anti-depressant medication being assessed by an expert interviewer. Pooled estimates were also examined by excluding extreme effect sizes and studies that did not adjust for baseline obesity or depression. Finally, analysis of the effect of potential unpublished studies was undertaken to evaluate the robustness of the meta-analysis. The funnel plot asymmetry was evaluated. However, we did compute Eggers regression and considered the studies to be asymmetrical if the intercept of Egger’s regression deviated from zero with *p* < 0.10 [49].

## Results

**Fig 1:**
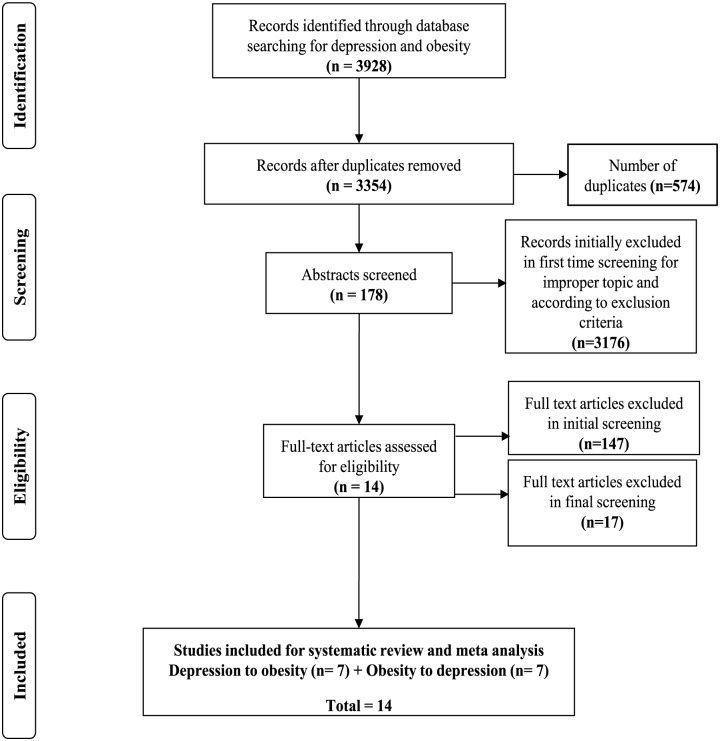
Flow diagram of the selection of articles through study. **from:** Moher D, Liberati A, Tetzlaff J, Altman DG, The PRISMA Group (2009). Preferred Reporting Items for Systematic Reviews and Meta-Analyses: The PRISMA Statement. PLoS Med 6(6): e1000097. doi:10.1371/journal.pmed1000097

A search of six electronic databases identified 3828 articles of which 3815 were excluded based on the criteria listed in Fig 1. Thirteen articles [8, 50–61] were included for systematic review and meta-analysis. All the included studies were prospective studies that examined the association between depression and obesity, of these, seven studies examined depression leading to obesity [8, 50, 51, 53, 54, 56, 61] and six studies investigated obesity leading to depression [52, 55, 57–60]. Three of the studies presented the longitudinal bidirectional data [52, 55, 61]. There were 32,026 participants available to investigate the possible link between these two conditions and other associated characteristics; these are summarized in Table 1.

**Table 1:** Characteristics of the included studies.

Author, year	Country	Study sample	Gender		Mean age (at baseline)	Follow-up duration (year)	Covariates	Assessment of depression	Assessment of BMI (baseline/follow-up)
			Male	Female					
**Depression leading to obesity**									
Pine et al., 1997	US	644	310	334	14	10	Physical health, smoking, social class, IQ, parent sociopathy, conduct disorder (6)	CD	S/S
Pine et al., 2001	US	177	-	-	11	15	Age, sex, cigarette use, alcohol use, social class, pregnancy, medication history (7)	CD	M/S
Goodman et al., 2002	US	9374	4656	4718	15	1	Age, sex, race, parental obesity, parent education, number parents at home, self-esteem, smoking, conduct disorder, physical activity, baseline BMI (11)	DS	S/S
Richardson et al., 2003 (34)	New Zealand	660	348	312	19	7	Childhood BMI, parental obesity, SES, maternal depression (4)	CD	M/M
Stice et al., 2005 (35)	US	496	-	496	13.5	4	Dietary restraint, compensatory behaviours, perceived parental obesity (3)	CD	M/M
Franko et al., 2005 (36)	US	1554	-	1554	16	5	Race, site, parent education, prior BMI (4)	DS	M/S
Anderson et al., 2010 (37)	US	918	-	918	12	2	Age, family income, time spent after school, self-esteem and physical activity (5)	DS	M/M
Kubzansky et al., 2012 (38)	US	1528	-	-	14.5	4	Age, race, parents education, pubertal development (4)	DS	M/M
Marmorstein et al., 2014 (43)	US	821	-	-	11	13	none	CD	M/M
**Obesity leading to depression**									
Goodman et al., 2002	US	9374	4656	4718	15	1	Age, sex, race, parental obesity, parent education, number parents at home, baseline CES-D score (7)	DS	S/-
Herva et al., 2006	Finland	3125	1159	1966	14	17	Father’s social class, family type, chronic diseases, smoking, alcohol use (5)	DS	S/-
Anderson et al., 2007	US	674	342	332	15	20	SES, race, smoking, parent psychopathology (4)	CD	S/-
Boutelle et al., 2010 (41)	US	488	-	488	13	4	Age, ethnicity (2)	CD	M/-
Frisco et al., 2013(42)	US	367	-	367	16	5	Age, ethnicity, family income, parents education, family structure, physical activity, pregnancy before follow-up wave 2 (7)	DS	M/-
Anderson et al., 2010 (37)	US	918	-	918	12	2	Age, family income, time spent after school, self-esteem and physical activity (5)	DS	M/-
Marmorstein et al., 2014 (43)	US	908	-	-	11	13	none	CD	M/M

Abbreviations: DS- Depressive symptoms, CD- Clinically diagnosed, S-self-reported, M- Measured. BMI- Body mass index, US-United States

### Depression leading to obesity

#### Study characteristics

The majority of the studies were from the US, with one study from New Zealand. There was considerable methodological variability. Both males and females were studied in three of the included studies [50, 52, 53] and only females were investigated in two other studies [8, 54]. Participants of the included studies were diverse in terms of race, ethnicity, social and other demographic characteristics. However, the predominance of caucasians in the samples should be noted. The sample size across studies ranged from 177 to 9374 with a mean age that varied between 11 and 19 years and with follow-up periods that ranged between 1 and 15 years. Depression was assessed by clinical diagnosis in five studies [8, 50, 51, 53, 61] with the remaining studies using depressive symptoms scales [54, 56]. At follow-up, self-reported information was used in three studies [50, 51, 54] whereas the others used measured weight and height to calculate BMI. To assess depressive symptoms/disorder a wide range of standard scales were used with a large set of covariates ranging from none to 11 across the studies.

#### Quantitative outcomes

A meta-analysis of the relative and absolute risk of obesity is presented in Table 2 (forest plot Figures C and D in S1 File). Overall, adolescents who were depressed at baseline were at a 70% (RR: 1.70, 95% CI: 1.40, 2.07) higher risk of obesity compared with those who did not experience depression. When the association was split by gender, no significant difference was found between males and females (*p* = 0.08). In terms of absolute risk, the baseline impact of depression contributed 4% (RD: 0.04, 95% CI: 0.01, 0.06) excess risk of obesity compared with non-depressed subjects.

**Table 2:** Pooled estimates of incidence depression leading to obesity measured in terms of relative and absolute risk using quality effect model.

Study	Obese*	No-obese	RR (95% CI)	Obese	No-obese	RD (95% CI)±	P-value (between groups)#
***Male***							
Pine et al., 1997	(9)/(50)	(34)/(260)	1.38 (0.70, 2.69)	(1)/(50)	(4)/(260)	0.00 (-0.04, 0.05)	***0*.*08***
Richardson et al., 2003	(2)/(30)	(33)/(445)	0.90 (0.37, 2.02)	(1/30)	(5)/(445)	0.02 (-0.04, 0.09)	
***Overall male***			**1.16 (0.68, 1.96)**			**0.00 (-0.02, 0.02)**	
***Female***							
Pine et al., 1997	(21)/(64)	(37)/(270)	2.39 (1.51, 3.80)	(3)/(64)	(4)/(270)	0.03 (-0.02, 0.09)	
Richardson et al., 2003	(13)/(37)	(59)/(391)	2.33 (1.29, 3.83)	(3)/(37)	(10)/(391)	0.06 (-0.03, 0.14)	
Stice et al., 2005	(1)/(11)	(18)/(443)	2.24 (0.63, 7.09)	(1)/(11)	(5)/(443)	0.08 (-0.09, 0.25)	
Franko et al., 2005	(197)/(622)	(115)/(932)	2.57 (1.18, 4.65)	(46)/(622)	(25)/(932)	0.05 (0.02, 0.07)	
Anderson et al., 2010	(38)/(131)	(108)/(787)	2.00 (0.93, 5.68)	(21)/(131)	(56)/(787)	0.09 (0.02, 0.15)	
***Overall female***			**2.34 (1.74, 3.15)**			**0.05 (0.03, 0.08)**	
***Both (not able to separate)***							
Pine et al., 2001	(13)/(90)	(7)/(87)	1.80 (0.75, 4.29)	(1)/(90)	(1)/(87)	0.00 (-0.03, 0.03)	
Goodman et al., 2002	(157)/(856)	(733)/(8518)	2.13 (1.03, 4.89)	(157)/(856)	(733)/(8518)	0.10 (0.07, 0.12)	
Kubzansky et al., 2012	(31)/(230)	(124)/(1298)	1.41 (1.13, 1.84)	(9)/(230)	(33)/(1298)	0.01 (-0.01, 0.04)	
Marmorstein et al., 2014	(15)/(97)	(110)/(724)	1.02 (0.38, 2.77)	(2)/(97)	(10)/(724)	0.01 (-0.02, 0.04)	
***Overall Both***			**1.48 (1.15, 1.91)**			**0.03 (-0.01, 0.08)**	
**Overall**			**1.70 (1.40, 2.07)**			**0.04 (0.01, 0.06)**	

Abbreviations: RR- relative risk, RD- risk difference, CI-confidence interval; ± Risk difference (RD) was measured based on estimated cumulative incidence at 1 year across the studies with follow-up of more than a year. This interpolation at 1 year had made difference in number of obese and non-obese to calculate risk difference (RD).

* Obesity was defined by WHO (World Health Organization) BMI cut-off—BMI≥ 30kg/m^2^ and non-obese defined BMI<30 kg/m^2^.

^#^ P-value determined the interaction effect between male and female was measured using RR and significance level set at <0.05

#### Sensitivity analysis and publication bias

A sensitivity analysis to assess the robustness of the association of depression leading to obesity was conducted and is presented in Table 3. None of the sub-groups were significantly different except for life stage, in which females during young adulthood, when compared with females in late adolescence, were found to be at increased risk of developing obesity (***p*** = 0.04). A leave-one-out sensitivity analysis did not show variance in pooled estimates. In addition, exclusion of the studies with outlying results did not significantly change the overall estimates.

**Table 3:** Sensitivity analyses of depression leading to obesity.

Sub-groups	Obesity (BMI≥30 kg/m^2^)*											
	Male				Female				Combined (male and female)			
	No of studies and sample size	RR (95%CI)	RD (95%CI)	p-value (between groups)#	No of studies and sample size	RR (95%CI)	RD (95%CI)	p-value (between groups)#	No of studies and sample size	RR (95%CI)	RD (95%CI)	p-value (between groups)#
**Risk in life-stages**												
Late Adolescent	-	-	-	-	2 (1414)	2.10 (1.01, 2.34)	0.05 (0.02, 0.07)	***0*.*04***	2 (1705)	1.58 (1.20, 2.07)	0.06 (-0.02, 0.14)	***0*.*64***
Young Adulthood	2 (658)	1.70 (1.40, 2.07)	0.01 (-0.02, 0.05)		3 (2200)	2.41 (2.24, 4.38)	0.09 (0.02, 0.15)		2 (10195)	1.33 (0.68, 2.59)	0.00 (-0.02, 0.03)	
**Follow-up duration**												
<10 years	1 (348)	0.90 (0.37, 2.02)	0.02 (-0.04, 0.09)	***0*.*44***	4 (3280)	2.33 (1.59, 3.38)	0.03 (-0.02, 0.09)	***0*.*37***	2 (1705)	1.58 (1.20, 2.07)	0.06 (-0.02, 0.14)	***0*.*64***
≥10 years	1 (310)	1.38 (0.70, 2.69)	0.00 (-0.04, 0.05)		1 (334)	2.39 (1.51, 3.80)	0.06 (0.03, 0.08)		2 (10195)	1.33 (0.68, 2.59)	0.00 (-0.02, 0.03)	
**Measures of Depression**												
Depressive symptoms	-	-	-	***-***	2 (2472)	2.33 (1.35, 4.03)	0.05 (0.00, 0.09)	***0*.*48***	2 (1705)	1.58 (1.20, 2.07)	0.06 (-0.01, 0.12)	***0*.*64***
Clinically diagnosed	2 (658)	1.70 (1.40, 2.07)	0.01 (-0.02, 0.05)		3 (1142)	2.34 (1.64, 3.34)	0.06 (0.02, 0.09)		2 (10195)	1.33 (0.68, 2.59)	0.00 (-0.03, 0.03)	
**Adjustment of confounders**												
Adjusted <5 confounders	1 (348)	0.90 (0.37, 2.02)	0.02 (-0.04, 0.09)	***0*.*44***	3 (2362)	2.40 (1.61, 3.59)	0.18 (0.11, 0.24)	***0*.*52***	2 (9551)	1.38 (1.09, 1.75)	0.03 (-0.01, 0.07)	***0*.*26***
Adjusted ≥ 5 confounders	1 (310)	1.38 (0.70, 2.69)	0.00 (-0.04, 0.05)		2 (1252)	2.30 (1.53, 3.47)	0.16 (0.10, 0.23)		2 (2349)	1.98 (1.11, 3.53)	0.09 (0.07, 0.12)	
**Region**												
US	1 (310)	1.38 (0.70, 2.69)	0.00 (-0.04, 0.05)	***0*.*22***	4 (3302)	2.34 (1.65, 3.32)	0.05 (0.03, 0.08)	***0*.*31***	4 (11900)	1.48 (1.15, 1.91)	0.03 (-0.01, 0.08)	***-***
Europe & others	1 (348)	0.90 (0.37, 2.02)	0.02 (-0.04, 0.09)		1 (312)	2.33 (1.59, 3.83)	0.06 (-0.03, 0.14)					

Abbreviation: RR- relative risk, RD- risk difference, CI- confidence interval and BMI-Body mass index; Depression was measured based on symptoms using different rating scales and clinically diagnosed

* BMI was defined by WHO (World Health Organization) cut off

^#^ P-value determined whether the difference between groups in each category of the sensitivity analysis was measured based on RR and significance level set at <0.05

Finally, the funnel plot (Figure A in S1 File) demonstrated asymmetry suggesting a clear bias (intercept on Eggers regression 2.83, *p*<0.001) in favour of studies with positive outcomes.

### Obesity leading to depression

#### Study characteristics

Studies reporting obesity as a predictor of depression were methodologically heterogeneous. The majority of the included studies were from the US (85%). Three studies [52, 57, 58] reported this association for both males and females and three other studies reported data only for females [55, 59, 60]. Participants were diverse in relation to age, race, ethnicity, social and demographic characteristics, although the majority of females were caucasian. Approximately 16000 participants (15854) were included in this investigation with sample sizes ranging from 367 to 9374 participants. The mean age varied from 11 to 15 years and follow-up time from 1 up to 20 years. For BMI at baseline, three studies [52, 57, 58] relied on self-reported information and the remainder of the studies used measured height and weight [55, 59–61]. At follow-up, four studies assessed depression using depressive symptoms [52, 55, 57, 60] and the rest of the studies used clinical diagnoses [58, 59, 61]. All but one of the studies [57] removed the baseline depressed participants to assess the incidence. To assess depressive symptom/disorder a wide range of standard scales were used and considered large set of covariates (range from none to 8) across the studies.

#### Quantitative outcomes

The overall pooled RR was 1.40 (RR: 1.40, 95% CI: 1.16, 1.70) for depression (Table 4, forest plot Figures E and F in S1 File). When the pooled results were stratified by gender, there were no statistically different results for males and females (***p*** = 0.18). In terms of absolute risk, subjects who were obese at baseline contributed 1% (RD: 0.01, 95%, CI: 0.00, 0.02) excess risk of depression compared with the non-obese.

**Table 4:** Pooled estimates of incidence obesity leading to depression measured in terms of relative and absolute risk using quality effect model.

Study	Depression*	No-depression	RR (95% CI)	Depression	No-depression	RD (95% CI)±	P-value (between groups)#
***Male***							
Herva et al., 2006	(28)/(177)	(124)/(982)	1.25 (0.89, 1.75)	(2)/(177)	(8)/(982)	0.00 (-00.1, 0.02)	***0*.*18***
Anderson et al., 2007	(4)/(32)	(26)/(310)	1.49 (0.55, 2.89)	(1)/(32)	(2)/(310)	0.02 (-0.04, 0.09)	
***Overall male***			**1.31 (0.94, 1.83)**			**0.01 (-0.01, 0.03)**	
***Female***							
Herva et al., 2006	(41)/(200)	(205)/(1766)	1.76 (1.14, 2.23)	(3)/(200)	(13)/(1766)	0.01 (-0.01, 0.02)	
Boutelle et al., 2010	(9)/(53)	(50)/(443)	1.50 (0.78, 2.88)	(3)/(53)	(14)/(443)	0.03 (-0.04, 0.09)	
Anderson et al., 2010	(32)/(143)	(132)/(775)	1.38 (0.91, 2.10)	(13)/(143)	(49)/(775)	0.03 (-0.02, 0.08)	
Anderson et al., 2007	(3)/(10)	(41)/(322)	2.36 (1.45, 4.95)	(1)/(10)	(3)/(322)	0.09 (-0.10, 0.28)	
Frisco et al., 2013	(61)/(514)	(306)/(4729)	1.83 (1.17, 3.09)	(13)/(514)	(63)/(4729)	0.01 (0.00, 0.03)	
***Overall female***			**1.72 (1.38, 2.15)**			**0.02 (0.00, 0.03)**	
***Both (not able to separate)***							
Goodman et al., 2002	(92)/(892)	(763)/(8482)	1.15 (0.81, 1.63)	(92)/(892)	(763)/(8482)	0.01 (-0.01, 0.03)	
Marmorstein et al., 2014	(19)/(173)	(115)/(735)	0.71 (0.34, 1.48)	(2)/(173)	(10)/(735)	0.00 (-0.02, 0.02)	
***Overall Both***			**0.97 (0.63, 1.48)**			**0.00 (-0.01, 0.02)**	
**Overall**			**1.40 (1.16, 1.70)**			**0.01 (0.00, 0.02)**	

Abbreviations: RR- relative risk, RD- risk difference, CI-confidence interval; ± Risk difference (RD) was measured based on estimated cumulative incidence at 1 year across the studies with follow-up of more than a year. This interpolation at 1 year had made difference in number of depressed and non-depressed to calculate risk difference (RD).

* Depression was measured based on symptoms using different rating scales and clinically diagnosed

^#^ P-value determined the interaction effect between male and female was measured using RR and significance level set at <0.05

#### Sensitivity analysis and publication bias

In sensitivity analyses, significant effect variation was observed for males and females using either measure of risk of depression (see Table 5). Females who were obese were at a higher risk of depression in young adulthood compared with late adolescence (*p* = 0.02). This association was stronger when the females were exposed for a longer period of time (more than 10 years) compared with shorter follow-ups (less than 10 years) (*p* = 0.03). The remaining sub-groups did not show any significant differences in estimates. Besides these analyses, robustness of the overall pooled estimate was also checked by excluding studies with outlying results and studies without measurement of depression at baseline but no noticeable changes were observed. Finally, leave-one-out sensitivity analyses were applied but did not make a significant difference to the overall pooled estimate.

**Table 5:** Sensitivity analyses of obesity leading to depression.

Sub-groups	Depression*											
	Male				Female				Combined (male and female)			
	No of studies and sample size	RR (95%CI)	RD (95%CI)	p-value (between groups)#	No of studies and sample size	RR (95%CI)	RD (95%CI)	p-value (between groups)#	No of studies and sample size	RR (95%CI)	RD (95%CI)	p-value (between groups)#
**Risk in life-stages**												
Late Adolescent	-	-	-	***-***	2 (1406)	1.40 (0.91, 1.76)	0.03 (-0.01, 0.07)	***0*.*02***	1 (9374)	1.15 (1.17, 1.92)	0.01 (-0.01, 0.03)	***0*.*06***
Young Adulthood	2 (1501)	1.31 (0.94, 1.83)	0.01 (-0.01, 0.03)		3 (2665)	1.89 (1.69, 2.68)	0.02 (0.00, 0.04)		1 (908)	0.71 (0.63, 1.48)	0.00 (-0.02, 0.02)	
**Follow-up duration**												
<10 years	-	-	-	***-***	3 (1773)	1.57 (1.13, 1.89)	0.02 (-0.02, 0.06)	***0*.*03***	1 (9374)	1.15 (1.17, 1.92)	0.01 (-0.01, 0.03)	***0*.*06***
≥10 years	2 (1501)	1.31 (0.94, 1.83)	0.01 (-0.01, 0.03)		2 (2298)	1.93 (1.76, 2.52)	0.02 (0.00, 0.03)		1 (908)	0.71 (0.63, 1.48)	0.00 (-0.02, 0.02)	
**Measures of Depression**												
Depressive symptoms	1 (1159)	1.25 (0.89, 1.75)	0.00 (-0.01, 0.02)	***0*.*5***	3 (3251)	1.66 (1.29, 2.15)	0.04 (-0.03, 0.11)	***0*.*67***	1 (9374)	1.15 (1.17, 1.92)	0.01 (-0.01, 0.03)	***0*.*06***
Clinically diagnosed	1 (342)	1.49 (0.55, 2.89)	0.02 (-0.04, 0.09)		2 (820)	1.86 (1.19, 2.92)	0.01 (0.00, 0.02)		1 (908)	0.71 (0.63, 1.48)	0.00 (-0.02, 0.02)	
**Adjustment of confounders**												
Adjusted <5 confounders	1 (342)	1.49 (0.55, 2.89)	0.02 (-0.04, 0.09)	***0*.*7***	2 (820)	1.91 (1.22, 2.98)	0.04 (-0.03, 0.11)	***0*.*63***	1 (908)	0.71 (0.63, 1.48)	0.00 (-0.02, 0.02)	***0*.*06***
Adjusted ≥ 5 confounders	1 (1159)	1.25 (0.89, 1.75)	0.00 (-0.01, 0.02)		3 (3251)	1.69 (1.31, 2.16)	0.01 (0.00, 0.02)		1 (9374)	1.15 (1.17, 1.92)	0.01 (-0.01, 0.03)	
**Region**												
US	1 (342)	1.49 (0.55, 2.89)	0.02 (-0.04, 0.09)	***0*.*7***	4 (2105)	1.70 (1.27, 2.27)	0.02 (0.00, 0.04)	***0*.*88***	2 (10282)	0.97 (0.63, 1.48)	0.00 (-0.01, 0.02)	***-***
Europe & others	1 (1159)	1.25 (0.89, 1.75)	0.00 (-0.01, 0.02)		1 (1966)	1.76 (1.14, 2.23)	0.01 (-0.01, 0.02)		-	-	-	

Abbreviation: RR- relative risk, RD- risk difference, CI- confidence interval

* Depression was measured based on symptoms using different rating scales and clinically diagnosed

^#^ P-value determined whether the difference exists between groups in each category of the sensitivity analysis was measured based on RR and significance level set at <0.05

The funnel plot (Figure B in S1 File) was found to be asymmetrical (intercept on Eggers regression 0.79, *p* <0.001) suggesting the presence of publication bias favouring studies with positive outcomes.

## Discussion

This is the first meta-analysis to assess the bi-directional associations between depression and obesity in adolescents. Our results showed a 70% increased risk of being obese in depressed adolescents; conversely obese adolescents had a 40% greater risk of being depressed. The association was statistically significant in terms of the depression and obesity link regardless of direction and when stratified by gender the bi-directional association was found for both males and females. In terms of impact, the risk difference was also bi-directional.

Our result confirms the bi-directional association between obesity and depression in both relative and absolute terms. This finding is in accordance with the results of a recent review which also identified a bi-directional link between obesity and depression [29]. However, this review included studies of both adolescents and children (less than 12 years old) and did not include age based sub-group analyses. Similarly, Muhlig et al. reported a weak association between obesity and depression in both directions [30]. However, they failed to ensure a strong conclusion due to the methodological variability of the included studies. By way of contrast the methodology used in our study can efficiently balance these methodological differences and has established a prospective link between obesity and depression.

Our meta-analysis has also confirmed the reciprocal associations between obesity and depression in both males and females. The strength of association was found to be stronger for females compared with males. This result is in line with recently published reviews which also indicate a gender effect in the bidirectional associations of obesity and depression with a stronger relationship being evident for females [29, 30]. Biologically females during puberty experience complex developmental processes that may result in the earlier onset of obesity disorders and which may persist throughout adolescence to adulthood [62, 63]. In the case of males, puberty occurs approximately two years later than in females [64]. This earlier pubertal development in females may also be associated with the marked gender differences in adolescent depression [65]. However, the gender difference in depression is also observed post-puberty, this is more closely tied to hormonal changes than to chronological age [66, 67] and this gender difference persisting across the female reproductive life course [68, 69]. Behavioural and lifestyle factors may also enhance this gender disparity [70, 71]. In one study it was found that females were dissatisfied with their bodies regardless of their actual weight, whereas males became concerned only when they were objectively overweight [72]. Females tend to more dissatisfied with their bodies than males [73]. Negative life events such as bullying as well as poor self-esteem [12] can produce stress, which may in turn increase the risk of obesity and depressive symptoms in adolescent females more so than in adolescent males [74, 75].

In sensitivity analysis, we found that the bi-directional association between obesity and depression was stronger for females in young adulthood than in late adolescence. It is likely that developmental factors are relevant across late adolescence and adulthood. The tracking of BMI from adolescence into adulthood is high [76, 77] particularly for females; for example, according to one study, BMI at age 18 predicts 50% of the BMI variance at age 35 years [76]. Similarly with depression, although there is little gender disparity in pre-adolescence, by mid-adolescence the female preponderance of depression extends across the female reproductive life course with the rate for females being approximately double that for males (OR: 1.7, CI:1.5–2.0) [68, 78]. These associations might also be due to the shifts in life stages that trigger both choices and challenges, including decisions about higher education, transition into the labour market, moving away from the family of origin and sometimes into marriage and parenthood [79]. A longer follow-up time (more than 10 years) was more strongly predictive of the reciprocal association between depression and obesity. This suggests that the longer a female is either obese or depressed, the more likely it is that lifestyle and environmental factors may enhance this association [51, 55].

Our funnel plot suggested possible publication bias which may result either from either clinical heterogeneity or methodological heterogeneity between studies. However, publication bias may also exist because the published studies may not be representative of all studies that have been performed because positive results tend to be submitted and published more often than negative results [80]. Finally, there may also be small study effects (smaller studies show stronger effects) that can cause the asymmetry in funnel plots [80].

The findings of this study are based only on longitudinal studies and a more rigorous methodology has been used to define more accurate measures of effect estimation than have been previously presented. This gives additional strength to this review. To assess the risk association the RR was used instead of OR to provide more realistic effect estimates [43]. Secondly, all the previous review articles have used only relative measures to examine the strength of association between obesity and depression. This approach can magnify risk associations because of the nature of the RR or OR (i.e. a large RR or OR can be associated with very small changes in absolute risk) and this cannot be translated into individual decisions because it does not typically take baseline risk into account. To facilitate translation of the findings the absolute measure of risk allowed us to account for the baseline risk and to provide an estimate of the actual risk contribution by both obesity and depression. Thirdly, we used a QE model [35–37]; this model is able to neutralise possible biases associated with methodological differences among the studies which was not possible using the more common analytical model, the random effect model, used in previous review studies [28, 81]. Overall, the determination of the effect sizes in our study has greater efficacy to measure the actual and exact strength of associations between obesity and depression in adolescents.

This study has a number of limitations. Firstly, the majority of the studies examined predominantly caucasian populations and females, consequently our results require confirmation in more diverse samples with different racial and ethnic backgrounds and gender balances before we can generalise the estimates. In addition, the majority of the studies were conducted in North America and Europe, again making the estimates less generalisable, particularly to the developing world where cultural attitudes and beliefs may be different regarding mental health and obesity. In addition, use of antidepressant agents may also have a role in the association between depressive symptoms and overweight [82]. Only two of the included studies had controlled for their use [51, 53]. The remaining studies failed to report subject’s antidepressant use. We cannot completely rule out the possible confounding influence of antidepressant use on the overall association of depression and obesity. Finally, it was not possible to evaluate the potential interactive relationship of this bidirectional association due to insufficient data.

## Conclusion

In summary, this meta-analysis has established the bi-directional association between obesity and depression. The strength of the association was found to be stronger in the direction of depression leading to obesity than for obesity leading to depression but this may be an artefact of length of follow-up of the studies included. Both males and females were at risk of obesity and depression, however; the association was commonly stronger for females and for females in young adulthood more so than late adolescence. This evidence has significant implications particularly for young females in clinical practice to the extent that intervention early on for both obese and depressed young women may inhibit the development of the reciprocal disorder.

## Supporting Information

S1 File **Table A:** PRISMA Checklist; **Table B:** PICOS criteria for inclusion and exclusion of studies; **Table C:** Quality checklist; **Table D:** Quality score; **Figure A:** Funnel plot for depression leading to obesity; **Figure B:** Funnel plot for obesity leading to depression; **Figure C:** Adolescent depression leading to obesity (forest plot expressed in RR); **Figure D:** Adolescent depression leading to obesity (forest plot expressed in RD); **Figure E:** Adolescent obesity leading to depression (forest plot expressed in RR); **Figure F:** Adolescent obesity leading to depression (forest plot expressed in RD).
(DOCX)
