# Supplementary material for: Prospective Associations between Depression and Obesity for Adolescent Males and Females- A Systematic Review and Meta-Analysis of Longitudinal Studies
Source: PLoS One. 2016 Jun 10;11(6):e0157240. doi: 10.1371/journal.pone.0157240 (PMC4902254; doi:10.1371/journal.pone.0157240)
Supplement: S1 File — Table A: PRISMA Checklist; Table B: PICOS criteria for inclusion and exclusion of studies; Table C: Quality checklist; Table D: Quality score; Figure A: Funnel plot for depression leading to obesity; Figure B: Funnel plot for obesity leading to depression; Figure C: Adolescent depression leading to obesity (forest plot expressed in RR); Figure D: Adolescent depression leading to obesity (forest plot expressed in RD); Figure E: Adolescent obesity leading to depression (forest plot expressed in RR); Figure F: Adolescent obesity leading to depression (forest plot expressed in RD). (DOCX) [file pone.0157240.s001.docx]

**Supplement Tables and Figures: (Tables A-D, Figures A-F)**

**Table A: PRISMA Checklist**

| **Section/topic** | **#** | **Checklist item** | **Reported on page #** |
| --- | --- | --- | --- |
| **TITLE** | | |  |
| Title | 1 | Identify the report as a systematic review, meta-analysis, or both. | 1 |
| **ABSTRACT** | | |  |
| Structured summary | 2 | Provide a structured summary including, as applicable: background; objectives; data sources; study eligibility criteria, participants, and interventions; study appraisal and synthesis methods; results; limitations; conclusions and implications of key findings; systematic review registration number. | 2 |
| **INTRODUCTION** | | |  |
| Rationale | 3 | Describe the rationale for the review in the context of what is already known. | 3 |
| Objectives | 4 | Provide an explicit statement of questions being addressed with reference to participants, interventions, comparisons, outcomes, and study design (PICOS). | 4 |
| **METHODS** | | |  |
| Protocol and registration | 5 | Indicate if a review protocol exists, if and where it can be accessed (e.g., Web address), and, if available, provide registration information including registration number. | NA |
| Eligibility criteria | 6 | Specify study characteristics (e.g., PICOS, length of follow-up) and report characteristics (e.g., years considered, language, publication status) used as criteria for eligibility, giving rationale. | 5-6 |
| Information sources | 7 | Describe all information sources (e.g., databases with dates of coverage, contact with study authors to identify additional studies) in the search and date last searched. | 5 |
| Search | 8 | Present full electronic search strategy for at least one database, including any limits used, such that it could be repeated. | 5 |
| Study selection | 9 | State the process for selecting studies (i.e., screening, eligibility, included in systematic review, and, if applicable, included in the meta-analysis). | 6 |
| Data collection process | 10 | Describe method of data extraction from reports (e.g., piloted forms, independently, in duplicate) and any processes for obtaining and confirming data from investigators. | 6 |
| Data items | 11 | List and define all variables for which data were sought (e.g., PICOS, funding sources) and any assumptions and simplifications made. | NA |
| Risk of bias in individual studies | 12 | Describe methods used for assessing risk of bias of individual studies (including specification of whether this was done at the study or outcome level), and how this information is to be used in any data synthesis. | Not available |
| Summary measures | 13 | State the principal summary measures (e.g., risk ratio, difference in means). | 6 |
| Synthesis of results | 14 | Describe the methods of handling data and combining results of studies, if done, including measures of consistency (e.g., I^2^) for each meta-analysis. | 6-7 |

| **Section/topic** | **#** | **Checklist item** | **Reported on page #** |
| --- | --- | --- | --- |
| Risk of bias across studies | 15 | Specify any assessment of risk of bias that may affect the cumulative evidence (e.g., publication bias, selective reporting within studies). | 8 |
| Additional analyses | 16 | Describe methods of additional analyses (e.g., sensitivity or subgroup analyses, meta-regression), if done, indicating which were pre-specified. | 8 |
| **RESULTS** | | |  |
| Study selection | 17 | Give numbers of studies screened, assessed for eligibility, and included in the review, with reasons for exclusions at each stage, ideally with a flow diagram. | 9; Figure 1 |
| Study characteristics | 18 | For each study, present characteristics for which data were extracted (e.g., study size, PICOS, follow-up period) and provide the citations. | 9-11, Table 1 |
| Risk of bias within studies | 19 | Present data on risk of bias of each study and, if available, any outcome level assessment (see item 12). | NA |
| Results of individual studies | 20 | For all outcomes considered (benefits or harms), present, for each study: (a) simple summary data for each intervention group (b) effect estimates and confidence intervals, ideally with a forest plot. | S3Fig, S4 Fig, S5Fig and S6 Fig |
| Synthesis of results | 21 | Present results of each meta-analysis done, including confidence intervals and measures of consistency. | Table 2 and Table 3 |
| Risk of bias across studies | 22 | Present results of any assessment of risk of bias across studies (see Item 15). | 10, 12, S1 Fig, S2 Fig |
| Additional analysis | 23 | Give results of additional analyses, if done (e.g., sensitivity or subgroup analyses, meta-regression [see Item 16]). | 10, 12, Table 4 and Table 5 |
| **DISCUSSION** | | |  |
| Summary of evidence | 24 | Summarize the main findings including the strength of evidence for each main outcome; consider their relevance to key groups (e.g., healthcare providers, users, and policy makers). | 13.-16 |
| Limitations | 25 | Discuss limitations at study and outcome level (e.g., risk of bias), and at review-level (e.g., incomplete retrieval of identified research, reporting bias). | 16-17 |
| Conclusions | 26 | Provide a general interpretation of the results in the context of other evidence, and implications for future research. | 17 |
| **FUNDING** | | |  |
| Funding | 27 | Describe sources of funding for the systematic review and other support (e.g., supply of data); role of funders for the systematic review. | NA |

*From:*  Moher D, Liberati A, Tetzlaff J, Altman DG, The PRISMA Group (2009). Preferred Reporting Items for Systematic Reviews and Meta-Analyses: The PRISMA Statement. PLoS Med 6(6): e1000097. doi:10.1371/journal.pmed1000097

**Table B:** PICOS criteria for inclusion and exclusion of studies

| **Parameter** | **Inclusion Criteria** | **Exclusion Criteria** |
| --- | --- | --- |
| Population | 1. Adolescent male and female  2. Study population are from developed countries | Men and women aged over 18 years |
| Exposure | 1. Overweight and obesity (leading depression)  2. Depression (leading overweight/obesity) | Weight change and other measures of mental health |
| Comparator | N/A | N/A |
| Outcomes | Odds and relative risk (RR) for either direction of association between depression and overweight/obesity | Hazard ratio and other measures |
| Study design | Prospective studies | Cross-sectional study, case-control study and randomized control trial  Editorials  Methodological articles |

**Table C:** Quality checklist

| Q1 | Design Specific Bias | Was the data completely prospectively collected? | No (0) |
| --- | --- | --- | --- |
|  |  |  | Yes (1) |
| Q2 | Study selection Bias | Was there significant refusal to participate in baseline data collection? | No (0) |
|  |  |  | Yes (1)= > 20% of prospective collection of baseline data or not reported |
| Q3 |  | Were the number of participants lost at each stage or protocol deviations, acceptable (based on reason for dropout) or <20%? | No or not reported (0) |
|  |  |  | Yes (1) |
| Q4 |  | Were the eligibility criteria clearly specified and uniformly applied? | No or no description (0) |
|  |  |  | In part (0.50) |
|  |  |  | Yes (1) |
| Q5 |  | Study population sufficiently describe (e.g. gender, age distribution) | No (0) |
|  |  |  | Yes (1) |
| Q6 |  | Sampling process sufficiently describe (sampling frame e.g. telephone directory, sampling method, sample size calculation) | No (0) |
|  |  |  | Yes (1) |
| Q7 |  | Characteristics of drop-outs or non-respondents reported | No (0) |
|  |  |  | Yes (1) |
| Q8 | Adjustment of Confounder | Number of confounder considered/adjusted in the analysis | >10 confounders =3  6-10 confounders = 2  1-5 confounders = 1  Unadjusted = 0 |
| Q9 | Information Bias | Was timing of outcome assessment and duration of follow-up adequate for outcomes to occur? | No (0) = <1 year |
|  |  |  | Yes (1) =  ≥ 1 year |
| Q10 |  | Psychological predictors assessed using valid and precise instrument | (0) = self-reported depressive symptoms |
|  |  |  | (1)= Clinically measured using valid instruments |
| Q11 |  | How accurately was exposure measured? | (0) - self-report (at any time point) |
|  |  |  | (1) - measured (at both time point) |
| Q13 |  | Adjustment for baseline depression/BMI within all relevant studies | (0) - no or not reported or not required |
|  |  |  | (1) - yes |
| Q14 | Statistical analysis | Was the analysis logically laid out and well conceptualized? | No (0) |
|  |  |  | Yes (1) |
| Q15 |  | Was missing data analysed using imputation methods? | No (0) |
|  |  |  | Yes (1) |

**Table D:** Quality score

| Studies | Design Specific Bias (1) | Selection Bias (6) | Confounder (3) | Information bias (4) | Statistical Methods (2) | Score |
| --- | --- | --- | --- | --- | --- | --- |
|  | **Obesity to depression** | | | | | |
| Herva et al., 2006 | 1 | 4.5 | 1 | 2 | 1 | 0.79 |
| Anderson et al., 2007 | **1** | 2.5 | 1 | 3 | 1 | 0.71 |
| Anderson et al., 2010 | 1 | 3 | 1 | 4 | 1 | 0.83 |
| Frisco et al., 2013 | 1 | 3 | 2 | 4 | 1 | 0.92 |
| Goodman et al., 2002 | 1 | 3 | 0 | 4 | 1 | 0.75 |
| Marmorstein et al., 2014 | 1 | 3 | 3 | 4 | 1 | 1.00 |
|  | **Depression to obesity** | | | | | |
| Pine et al., 1997 | 1 | 5 | 1 | 3 | 1 | 0.92 |
| Pine et al., 2001 | 1 | 4 | 0 | 3 | 1 | 0.75 |
| Goodman et al., 2002 | 1 | 3 | 3 | 4 | 1 | 1.00 |
| Richardson et al., 2003 | 1 | 2.5 | 1 | 4 | 2 | 0.87 |
| Stice et al., 2005 | 1 | 4 | 1 | 4 | 1 | 0.92 |
| Franko et al., 2005 | 1 | 5 | 1 | 3 | 1 | 0.92 |
| Anderson et al., 2010 | 1 | 3 | 1 | 4 | 1 | 0.83 |
| Kubzansky et al., 2012 | 1 | 4 | 1 | 3 | 1 | 0.83 |
| Marmorstein et al., 2014 | 1 | 3 | 3 | 4 | 1 | 1.00 |

- **Total quality score-16**

**Funnel plot**

**Fig A:** Funnel plot for depression leading to obesity **Fig B:** Funnel plot for obesity leading to depression

**Fig C**: Adolescent depression leading to obesity (forest plot expressed in RR)

**Fig D**: Adolescent depression leading to obesity (forest plot expressed in RD)

**Fig E**: Adolescent obesity leading to depression (forest plot expressed in RR)

**Fig F**: Adolescent obesity leading to depression (forest plot expressed in RD)
